# Same-sex behavior disclosure to health care providers associated with greater awareness of pre-exposure prophylaxis

**DOI:** 10.1186/s12889-021-12317-z

**Published:** 2021-12-10

**Authors:** Wangnan Cao, Xinyi You, Jinghua Li, Liping Peng, Jing Gu, Chun Hao, Fengsu Hou, Dannuo Wei, Yang Deng, Yuantao Hao, Phoenix Kit-han Mo

**Affiliations:** 1 Department of Social Medicine and Health Education, School of Public Health, Peking University, Beijing, China; 2 School of Public Health, Sun Yat-Sen University, Guangzhou, China; 3 Sun Yat-sen Global Health Institute, Sun Yat-sen University, Guangzhou, China; 4 Department of Public Mental Health, Kangning Hospital, Shenzhen, Guangdong China; 5 Jockey Club School of Public Health and Primary Care, The Chinese University of Hong Kong, Ngan Shing St, Shatin, Hong Kong

**Keywords:** Men who have sex with men, Same-sex behavior disclosure, Health care provider, HIV testing, U=U, PrEP

## Abstract

**Background:**

This study aimed to determine whether the disclosure of same-sex behavior to health care providers (HCPs) is associated with higher rates of prior human immunodeficiency virus (HIV) testing experience and greater awareness of immediate antiretroviral therapy (ART), Undetectable = Untransmittable (U=U), and pre-exposure prophylaxis (PrEP) among men who have sex with men (MSM).

**Methods:**

We conducted a cross-sectional survey among 689 adult males in Chengdu, China who self-reported having had anal intercourse with at least one man in the past 6 months. We measured same-sex behavior disclosure to three types of HCPs (hospital clinicians, community-based organization peer educators, and Center for Disease Control and Prevention public health specialists), and the awareness of immediate ART, U=U, and PrEP.

**Results:**

Of the 689 enrolled participants, 31.4% had disclosed their same-sex behavior to some or all of the clinicians, 83.9% had done so to the peer educators, and 56.8% had done so to the public health specialists. Approximately four in five (82.1%) of the participants had ever been tested for HIV. The awareness rate was 84.8% for immediate ART, 20.2% for U=U, and 50.7% for PrEP. After controlling for significant background variables, same-sex behavior disclosure to clinicians was associated with greater awareness of PrEP (adjusted odds ratio [AOR] = 1.64; 95% confidence interval [CI]: 1.08–2.48), but similar findings were not reported regarding disclosure to peer educators or public health specialist. Same-sex behavior disclosure to any types of HCPs was not associated with HIV testing experience, and awareness of immediate ART or U=U.

**Conclusions:**

The rates of same-sex behavior disclosure varied with different types of HCPs. Disclosure to clinicians was associated with greater awareness of PrEP, but not awareness of immediate ART or U=U.

## Background

In 2019, key populations (including men who have sex with men [MSM], people who inject drugs, sex workers, transgender people and prisoners) and their partners accounted for 62% of all new HIV infections globally [1]. In contrast to a decreasing trend in HIV incidence in people who inject drugs and in sex workers, HIV infections in MSM increased by 25% between 2010 and 2019 [1]. Moreover, high HIV prevalence and incidence rates have been continuing among MSM in most countries [2]. One recent review of 25 studies reported that the HIV incidence among Chinese MSM was 5.61 per 100 person-years and found that the incidence increased over the study period from 2005 to 2014 [3].

MSM are likely to face substantial stigma and discrimination [4]. Those who disclose their same-sex behavior to others may experience many negative consequences, such as the end of relationships, the loss of employment, and violence [5, 6]. Disclosure of same-sex behavior varies across countries due to variations in the levels of stigma and access to health and counselling services, and differences in sociocultural backgrounds [7]. Minority Stress Theory reveals that minority group, such as MSM, suffer from chronic stress related to a lack of social identity, resulting in self-stigma and stigma from society, which are prominent determinants of poor mental health [8]. Considering that homosexuality remains illegal or highly stigmatized in China, Chinese MSM are less likely to disclose their sexuality than their counterparts in Western countries, who enjoy more supportive environments and policies [9–11].

Despite the potential negative consequences associated with disclosure, it is recommended to disclose their same-sex behavior to health care providers (HCPs) to facilitate access to HIV prevention and treatment services, and to receive tailored care that meets personal needs. There is evidence that the disclosure of same-sex behavior to HCPs facilitates discussion of HIV-related issues [12] and prompts HCPs to recommend HIV testing [13]. In contrast, if HCPs are unaware of their patients’ same-sex behavior, they may overlook their healthcare needs and fail to recommend appropriate HIV-preventive strategies. However, disclosure to HCPs is challenging for MSM, and thus the overall rates of disclosure vary from 16 to 90%: in China they vary from 16 to 24%, while in the United States, they vary from 44 to 90%. The disclosure rate is affected by race, setting, and a HCP’s environment [10]. For example, Qiao et al. reported that MSM were more willing to disclose their same-sex behaviour to HCPs in clinics with gay-friendly signs compared to those without these signs, and gay-friendly physicians were perceived to be safer to disclosure to than general practitioners [10].

The most recent and significant advances in HIV prevention and treatment are immediate antiretroviral therapy (ART), treatment as prevention (also known as Undetectable = Untransmittable [U=U]), and pre-exposure prophylaxis (PrEP). Briefly, immediate ART refers to starting ART immediately upon receiving a diagnosis of HIV infection, regardless of CD4+ count, and results in significant health benefits for patients living with HIV [14]. The U=U indicates that there is no risk of sexual transmission of HIV when the viral load is undetectable [15]. PrEP is a strategy for HIV prevention for people at high risk of infection and uses a single-pill regimen of antiretroviral drugs. This strategy can significantly reduce the risk of HIV transmission between HIV-discordant partners [16]. To end the HIV epidemic, it is recommended that HIV testing is first performed in all people at risk of infection, followed by immediate ART for those who tested positive, with ART continuing until an undetectable viral load is reached, or followed by PrEP for those who are HIV negative but at high risk of HIV infection [17, 18].

Awareness of these key strategies is fundamental to increasing their uptake. HCPs play a key role in increasing awareness amongst MSM by directly delivering the relevant information to MSM [19]. This is particularly important in the Chinese context, in which HIV and sexual minority groups are not openly discussed in public, whereas HCPs are widely accessible and regarded as credible [20, 21]. In China, there are typically three types of HCPs that provide HIV-related services to MSM, with these HCPs focused on different domains [22]. First, hospital clinicians serve people with a diagnosis or a risk of HIV infection by providing medical consultations. Second, peer educators at community-based organizations (CBOs) are community leaders who serve minorities, including MSM, and some of these educators are gay men. Third, public health specialists at the Chinese Center for Disease Control and Prevention (CDC) serve people with any potential HIV-related problems, and provide case confirmation, contact tracing, health education, and referral services. Thus, these three types of HCPs provide complementary HIV prevention and treatment services to MSM in China. Many CBOs and research groups design and deliver intervention programmes targeting MSM, either independently or under the leadership of the government sectors. Peer educators with great influence on and affinity with their peers are recruited by CBOs and are well-trained by public health specialists. These peer educators help disseminate knowledge about sexual behavior, HIV counselling and HIV testing for MSM populations through social media platforms and communication groups [23].

There is sufficient evidence in the literature documenting the associations between the disclosure of same-sex behavior to HCPs and the outcomes of HIV testing and immediate ART. However, there is insufficient evidence to determine whether the disclosure of same-sex behavior to HCPs is associated with awareness the U=U or PrEP. Such studies are particularly rare in the Chinese setting [10, 24, 25]. Another limitation is that HCPs, the disclosure recipients, have mostly been unclearly defined and studied as one group, without consideration of the different roles that various types of HCPs may play when MSM are their patients or clients.

Our objectives were to quantify the proportion of a Chinese sample of MSM who had disclosed their same-sex behavior to each of the three types of HCPs, and to determine the potential association between this disclosure and HIV testing, and awareness on immediate ART, U=U, and PrEP.

## Methods

### Study design and setting

We conducted a cross-sectional survey between November 2018 and April 2019 of MSM living in urban areas of Chengdu, China. MSM living in Chengdu are greatly affected by HIV, with the prevalence reported to be approximately 7.5%, compared to the national prevalence of 5.7% in China [26]. The large population of MSM in Chengdu means it is a key city in the emerging MSM epidemic, as it attracts a large number of migrants and it is a major stop on the heroin trafficking route through China. Therefore, heroin is more accessible in Chengdu than in other parts of China [27]. MSM who are drug users (e.g., heroin or crystal methamphetamine users) tend to have a greater amount of unprotected sex and more severe mental disorders than MSM who do not use drugs, which increases the risk of HIV infection in drug-using MSM and leads to worse health outcomes [28, 29].

### Participants and recruitment

Participants were recruited from clients of a local lesbian, gay, bisexual and transgender (LGBT)-friendly CBO that provides HIV-related services. Two staff members of the CBO contacted all potential participants from the CBO’s list of clients by phone to screen for their eligibility. The clients were eligible to participate if they were i) male at birth, ii) 18 years or older, iii) self-reported having had anal intercourse with at least one man in the past 6 months, and iv) had access to at least one HCP during their lifetime. No one was excluded based on HIV status. We identified 868 individuals that were eligible for participation in the study, and all of them were invited to attend the CBO to complete a questionnaire. During the study period, 689 participants (response rate of 79.4%) completed the survey in person. A research assistant was stationed at the CBO during the study period for progress monitoring, data collection and quality control.

Participants were briefed about the study purpose and procedure, and they provided written informed consent before starting the survey questionnaire, which was anonymous and self-administered using an iPad. The questionnaire took an average of 28 (standard deviation [SD] = 20) minutes to complete, and participants were offered USD7 in cash to compensate for the time taken to participate in the study. Confirmatory HIV testing was offered to all participants, for which separate consent was obtained. Ethical approval was obtained from the Ethics Committee of Sun Yat-sen University ([2018] 049).

### Measures

All questions used in the survey were pilot-tested on 43 eligible participants, who were excluded from the formal survey. Minor revisions were made based on the pilot results and the comments from participants.

#### Background

The following socio-demographic information was collected: age, ethnicity, local household registration (hukou), educational level, relationship status, employment status, personal income, and self-rated health status. We also asked the participants about their self-identified sexual orientation and self-reported HIV status.

#### Same-sex behavior disclosure to HCPs

Information on same-sex behavior disclosure to the three types of HCPs (hospital clinicians, CBO peer educators, and CDC public health specialists) was collected separately. An example question was: ‘Have you disclosed your same-sex behavior to the hospital clinicians who provided services to you?’ The response options included: ‘all of them’, ‘some of them’, ‘none of them’, and ‘not applicable to me’ (the latter response was given by participants who had not had access to any hospital clinicians during his lifetime). Participants who responded with ‘all of them’ or ‘some of them’ were combined and classified as Y = 1, while those who responded with ‘none of them’ were classified as Y = 0. Participants who responded with ‘not applicable to me’ were excluded from the analyses.

#### HIV testing history

We asked the participants if they had their HIV status tested before the survey (lifetime HIV testing) and also if they tested HIV in the past 6 months (recent HIV testing). The response options were ‘yes’ or ‘no’.

#### Sexual behaviors

Participants were asked to recall the total number of partners with whom they had had sex in the past month. Participants who reported having had sex with more than one partner in the past month were classified as having “multiple sexual partnerships”. Participants who did not use condoms with all partners in the past month were classified into “inconsistent condom use”.

#### Immediate ART awareness

We asked the participants to determine if the statement ‘A person who is newly diagnosed with HIV should start ART immediately’ was correct. The response options were ‘correct’, ‘incorrect’, and ‘I don’t know’. Those who responded with ‘incorrect’ or “I don’t know” were combined and classified as unawareness of immediate ART.

#### U=U awareness

We asked the participants to determine if the statement ‘A person with an undetectable viral load cannot transmit HIV to others’ was correct. The response options were ‘correct’, ‘incorrect’, and ‘I don’t know’. Those who responded with ‘incorrect’ or ‘I don’t know’ were combined and classified as unawareness of U=U.

#### PrEP awareness

Participants were asked if they had heard of any type of PrEP (daily oral PrEP, on-demand oral PrEP, or long-acting injectable PrEP), although none of these PrEP options were available in China at the time of the survey. Participants who answered ‘yes’ were classified as having PrEP awareness, whereas those who did not were classified as having no PrEP awareness.

### Statistical analysis

Bivariate associations were assessed using binary logistic regression to examine same-sex behavior disclosure to each of the three types of HCPs, with the four outcomes being HIV testing, immediate ART awareness, U=U awareness, and PrEP awareness. Bivariate analyses were performed to assess the association between background variables (e.g., age, educational level, income, self-identified sexual orientation, and sexual behaviors) and the four outcomes described above. The measures of association are presented as unadjusted odds ratios (ORu) with 95% confidence intervals (95% CIs). Subsequently, variables found to have a significant effect (*P* < 0.05) in the bivariate analyses were included in a multivariable logistic regression analysis, and the measures of association are presented as adjusted odds ratios (AORs) with 95% CIs. All statistical analyses were performed using SPSS Statistics (version 26; IBM, Armonk, NY, USA), and a two-tailed *P* value < 0.05 was considered statistically significant.

## Results

### Descriptive characteristics

The characteristics of the participants are presented in Table 1. Approximately half (45.6%) of the participants were 25 years old or younger and more than half (53.8%) were single. The majority (70.7%) of participants had a university-level education or above. Most participants self-reported as homosexual (76.2%), while 19.2% self-reported as bisexual (Table 1).

**Table 1:** Backgrounds, HIV testing history, and awareness among the participants (*N* = 689)

Items	N	%
**Backgrounds**		
Age (years)		
≤ 25	314	45.6
> 25	375	54.4
Ethnicity		
Han	668	97.0
Others	21	3.0
Local residence		
No	519	75.3
Yes	170	24.7
Highest education obtained		
Below than university	202	29.3
University or above	487	70.7
Relationship status		
Single	371	53.8
Married to a woman	92	13.4
Having boyfriends	176	25.5
Divorced/widow/others	50	7.3
Employment status		
Full time	441	64.0
Part time	29	4.2
Unemployed	219	31.8
Personal monthly income (USD)		
< 423	268	38.9
423–845	269	39.0
> 845	152	22.1
Self-identified sexual orientation		
Homosexual	525	76.2
Heterosexual	2	0.3
Bisexual	132	19.2
Other	30	4.4
Sell-reported HIV status		
Positive	21	3.0
Negative	537	77.9
Unknown	131	19.0
Self-rated health		
Very good/Good	468	67.9
In general	203	29.5
Very bad/Bad	18	2.6
**HIV testing**		
HIV testing ever (yes)	566	82.1
**Awareness**		
Immediate ART awareness	584	84.8
U=U awareness	139	20.2
PrEP awareness	349	50.7

*ART* Antiretroviral therapy, *U=U* Undetectable = Untransmittable, *PrEP* Pre-exposure Prophylaxis

**Fig. 1:**
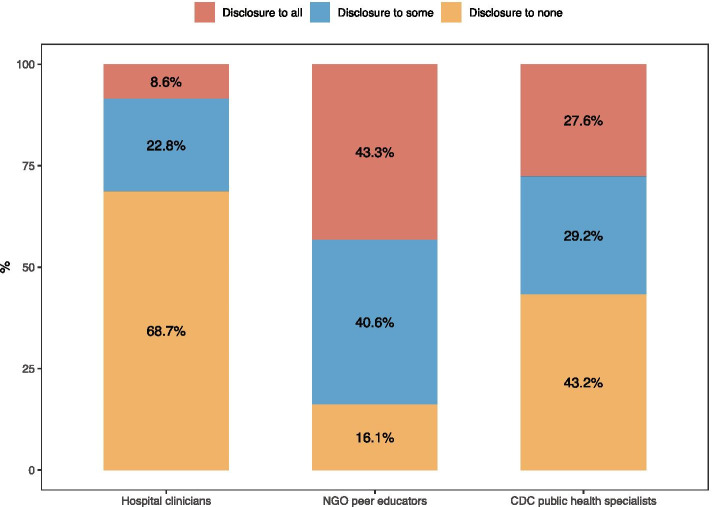
Same-sex behavior disclosure to different types of health care professionals

The rates of same-sex behavior disclosure varied by types of HCPs. Specifically, 79.7% (549/689) of the participants had prior experiences connecting with clinicians, 72.6% (500/689) had prior experiences connecting with public health specialists, and 98.0% (675/689) had prior experiences connecting with peer educators. Most participants (83.9%) had disclosed their same-sex behavior to at least one CBO peer educator, 56.8% had done so to at least one CDC public health specialist, and 31.4% had done so to at least one hospital clinician. Full disclosure (defined as ‘disclosure to all’) for a specific type of HCP was 43.3% for CBO peer educators, 27.6% for CDC public health specialists, and 8.6% for hospital clinicians (Fig. 1). These three disclosure behaviors were positively correlated with each other (Pearson correlation coefficients ranges: 0.22–0.43, all *P* < 0.001) (Table 2).

**Table 2:** Correlation analysis between three types of same-sex behavior disclosure

Items	Same-sex behavior disclosure to clinicians	Same-sex behavior disclosure to peer educators	Same-sex behavior disclosure to public health specialists
Same-sex behavior disclosure to clinicians	1.000		
Same-sex behavior disclosure to peer educators	0.216***	1.000	
Same-sex behavior disclosure to public health specialists	0.430***	0.314***	1.000

***P < 0.001

Approximately four in five (82.1%) of the participants had been tested for HIV at least once during their lifetime and half (50.6%) tested in the past 6 months. One-fifth (21.0%) of the participants had no sexual partner in the past month, 48.2% had sex with one partner, and 30.7% had multiple partners. Very few (3.0%) self-reported as HIV-positive, 77.9% self-reported as HIV-negative, and 19.0% self-reported as not knowing their HIV status. The majority (84.8%) of participants knew about immediate ART, half (50.7%) of the participants knew about PrEP, and one-fifth (20.2%) knew about U=U.

### Association between background variables and four outcome variables

As shown in Table 3, participants were more likely to have been tested for HIV if they were older (ORu = 2.62; 95% CI: 1.76–3.91; *P* < 0.001), had a higher income (423–845 USD vs. < 423 USD, ORu = 2.85; 95% CI: 1.78–4.56; *P* < 0.001). Participants who had multiple sexual partnership (ORu = 1.70; 95% CI: 1.07–2.70; *P* < 0.05) and used condom consistently (ORu = 2.04; 95% CI: 1.28–3.25; *P* < 0.01) were also more likely to be ever tested HIV. Compared to participants who were working full-time, unemployed participants were less likely to have been tested for HIV (ORu = 0.33; 95% CI: 0.22–0.50; *P* < 0.001).

**Table 3:** Bivariate regression analyses of the backgrounds associated with HIV testing and awareness on immediate ART, U=U, and PrEP

Items	HIV testing	Immediate ART awareness	U=U awareness	PrEP awareness
	ORu (95% CI)	ORu (95% CI)	ORu (95% CI)	ORu (95% CI)
**Sociodemographic**				
Age (years)				
≤ 25	1.00	1.00	1.00	1.00
> 25	2.62 (1.76, 3.91)***	1.01 (0.66, 1.54)	1.11 (0.76, 1.62)	0.92 (0.68, 1.24)
Ethnicity				
Minorities	1.00	1.00	1.00	1.00
Han	2.38 (0.95, 6.03)†	0.92 (0.27, 3.20)	0.62 (0.24, 1.63)	0.76 (0.32, 1.84)
Local residence				
No	1.00	1.00	1.00	1.00
Yes	1.20 (0.75, 1.92)	1.06 (0.65, 1.72)	0.94 (0.62, 1.45)	0.88 (0.62, 1.24)
Highest education obtained				
Below than university	1.00	1.00	1.00	1.00
University or above	1.03 (0.70, 1.52)	0.78 (0.51, 1.20)	0.63 (0.43, 0.92)**	1.95 (1.43, 2.64)***
Relationship status				
Single	1.00	1.00	1.00	1.00
Married to a woman	0.70 (0.40, 1.22)	0.83 (0.93, 0.49, 1.77)	1.21 (0.70, 2.07)	0.41 (0.25, 0.67)***
Having boyfriend	0.83 (0.52, 1.31)	0.75 (0.46, 1.21)	0.76 (0.48, 1.22)	1.75 (1.21, 2.53)**
Employment status				
Full time	1.00	1.00	1.00	1.00
Part time	0.52 (0.20, 1.35)	**0.45 (0.19, 1.07)†**	2.24 (1.00, 4.99)*	0.67 (0.31, 1.43)
Unemployed	0.33 (0.22, 0.50)***	1.01 (0.64, 1.56)	1.10 (0.73, 1.65)	0.95 (0.69, 1.32)
Personal monthly income (USD)				
< 423	1.00	1.00	1.00	1.00
423–845	2.85 (1.78, 4.56)***	0.97 (0.59, 1.61)	0.68 (0.43, 1.16)	0.99 (0.69, 1.42)
> 845	2.86 (1.74, 4.70)***	0.97 (0.58, 1.64)	0.88 (0.56, 1.38)	1.31 (0.90, 1.91)
Self-identified sexual orientation				
Homosexual	1.00	1.00	1.00	1.00
Bisexual	0.72 (0.45, 1.15)	0.98 (0.57, 1.67)	1.34 (0.85, 2.11)	0.42 (0.28, 0.63)***
Heterosexual/Others	0.87 (0.35, 2.18)	0.62 (0.26, 1.49)	0.97 (0.39, 2.42)	0.63 (0.31, 1.29)
Sell-reported HIV status				
Unknown	n.a.	1.00	1.00	1.00
Positive		1.42 (0.39, 5.18)	1.05 (0.32, 3.40)	1.22 (0.48, 3.09)
Negative		1.41 (0.86, 2.32)	1.16 (0.71, 1.90)	1.90 (1.29, 2.81)**
Multiple sex partnership				
No	1.00	1.00	1.00	1.00
Yes	1.70 (1.07, 2.70)*	1.40 (0.87, 2.25)	1.26 (0.85, 1.88)	0.89 (0.64, 1.23)
Consistent condom use				
No	1.00	1.00	1.00	**1.00**
Yes	2.04 (1.28, 3.25)**	0.76 (0.45, 1.27)	1.21 (0.77, 1.91)	1.49 (1.04, 2.12)*

†*P* < 0.10, *P < 0.05, **P < 0.01, ***P < 0.001

*ORu* univariate odds ratio, *n.a.* Not Applicable, *ART* Antiretroviral therapy, *U=U* Undetectable = Untransmittable, *PrEP* Pre-exposure Prophylaxis

Employment status was associated with awareness of the U=U, but in contrast to the results of HIV testing, participants who were working part-time were more likely to be aware of U=U (ORu = 2.24; 95% CI: 1.00–4.99; *P* < 0.05). Participants with a higher educational level were less likely to be aware of U=U (ORu = 0.63; 95% CI: 0.43–0.92; *P* < 0.01; Table 3).

Participants were more likely to be aware of PrEP if they had a higher educational level (ORu = 1.95; 95% CI: 1.43–2.64; *P* < 0.001), were in a stable relationship with a boyfriend (ORu = 1.75; 95% CI: 1.21–2.53; *P* < 0.01), were HIV-negative (ORu = 1.90; 95% CI: 1.29–2.81; *P* < 0.01), or used condom consistently (ORu = 1.49; 95% CI: 1.04–2.12; *P* < 0.05). Compared to participants who identified as homosexual, those who identified as bisexual were less likely to be aware of PrEP (ORu = 0.42; 95% CI: 0.28–0.63; *P* < 0.001; Table 3).

### Association between same-sex behavior disclosure and HIV testing

Same-sex behavior disclosure to public health specialists was associated with HIV testing in bivariate analysis (ORu = 1.80; 95% CI: 1.13–2.86; *P* < 0.01) but not associated in adjusted multivariable analysis (AOR = 1.47; 95% CI: 0.95–2.59; *P* < 0.10). Besides, same-sex behavior disclosure to clinicians or peer educators was not associated with HIV testing in either bivariate or adjusted analyses (Tables 4 and 5).

**Table 4:** Bivariate regression analyses of same-sex behavior disclosure associated with HIV testing and awareness on immediate ART, U=U, and PrEP

Items	HIV testing	Immediate ART awareness	U=U awareness	PrEP awareness
	ORu (95% CI)	ORu (95% CI)	ORu (95% CI)	ORu (95% CI)
Same-sex behavior disclosure to clinicians				
None disclosure	1.00	1.00	1.00	1.00
Partial/Full disclosure	**1.46 (0.89, 2.39)†**	1.08 (0.66, 1.76)	0.94 (0.60, 1.46)	1.85 (1.28, 2.67)***
Same-sex behavior disclosure to peer educators				
None disclosure	1.00	1.00	1.00	1.00
Partial/Full disclosure	0.90 (0.52, 1.56)	1.12 (0.64, 1.95)	**1.47 (0.90, 2.56)†**	1.16 (0.77, 1.75)
Same-sex behavior disclosure to public health specialists				
None disclosure	1.00	1.00	1.00	1.00
Partial/Full disclosure	1.80 (1.13, 2.86)**	0.85 (0.53, 1.37)	1.03 (0.67, 1.60)	1.46 (1.02, 2.08)*

†*P* < 0.10, *P < 0.05, **P < 0.01, ***P < 0.001;

*ORu* univariate odds ratio, *ART* Antiretroviral therapy, *U=U* Undetectable = Untransmittable, *PrEP* Pre-exposure Prophylaxis

**Table 5:** Multivariable regression analyses of same-sex behavior disclosure associated with HIV testing and awareness on immediate ART, U=U, and PrEP

Items	HIV testing	Immediate ART awareness	U=U awareness	PrEP awareness
	AOR (95% CI)	AOR (95% CI)	AOR (95% CI)	AOR (95% CI)
Same-sex behavior disclosure to clinicians				
None disclosure	1.00	1.00	1.00	1.00
Partial/Full disclosure	1.33 (0.74, 2.42)	1.10 (0.67, 1.80)	0.96 (0.61, 1.50)	1.64 (1.08, 2.48)*
Same-sex behavior disclosure to peer educators				
None disclosure	1.00	1.00	1.00	1.00
Partial/Full disclosure	0.83 (0.48, 2.10)	1.11 (0.64, 1.94)	1.49 (0.85, 2.61)	1.28 (0.80, 2.04)
Same-sex behavior disclosure to public health specialists				
None disclosure	1.00	1.00	1.00	1.00
Partial/Full disclosure	**1.47 (0.95, 2.59)**†	0.85 (0.53, 1.37)	1.07 (0.69, 1.65)	**1.42 (0.94, 2.14)**†

†*P* < 0.10, *P < 0.05, **P < 0.01, ***P < 0.001;

*AOR* adjusted odds ratio, variables that presented a statistically significant level (*P* < 0.05) were controlled in these multivariate regression analyses, *ART* Antiretroviral therapy, *U=U* Undetectable = Untransmittable, *PrEP* Pre-exposure Prophylaxis

### Association between same-sex behavior disclosure and immediate ART awareness or U=U awareness

Same-sex behavior disclosure to any of the three types of HCPs was not associated with awareness of immediate ART or U=U in either bivariate or adjusted analyses (Tables 4 and 5).

### Association between same-sex behavior disclosure and PrEP awareness

Same-sex behavior disclosure to clinicians was associated with PrEP awareness in both bivariate (ORu = 1.85; 95% CI: 1.28–2.67; *P* < 0.001) and adjusted analyses (AOR = 1.64; 95% CI: 1.08–2.48; *P* < 0.05). However, same-sex behavior disclosure to public health specialists was associated with PrEP awareness in bivariate analysis (ORu = 1.46; 95% CI: 1.02–2.08; *P* < 0.05) but not significant in adjusted multivariable analysis. Besides, same-sex behavior disclosure to peer educators was not associated with PrEP awareness in either bivariate or adjusted analyses (Tables 4 and 5).

## Discussion

This study investigated same-sex behavior disclosure to HCPs in a Chinese sample of MSM, and its association with HIV testing and with awareness of immediate ART, U=U, PrEP. We found that the rates of same-sex behavior disclosure varied by different types of HCPs. Disclosure to clinicians was associated with greater PrEP awareness. Disclosure to public health specialists was associated with higher rates of lifetime HIV testing and greater PrEP awareness in bivariate analyses. However, disclosure to HCPs, regardless of the type of provider, was not associated with awareness of immediate ART or U=U.

The rates of same-sex behavior disclosure varied by different types of HCPs, with disclosure rates to clinicians generally being low, those to peer educators generally being high, and those to public health specialists generally being medium. The low disclosure rates to clinicians may be attributable to MSM fearing discrimination and stigma from clinicians, avoiding unfair treatment in healthcare settings, and/or being concerned about breaches of confidentiality [9, 30]. High disclosure rates to peer educators may be attributable to the high level of trust MSM have in their peer educators and the provision of easily accessible HIV-related services by CBOs. However, disclosure rates to peer educators might be overestimated due to sample bias, as this MSM sample was recruited through a LGBT-friendly CBO. Clients of this CBO might be more likely than those who were not clients to disclose their same-sex behavior to the peer educators at the CBO.

Clinicians, peer educators, and public health specialists play equally important roles in providing HIV prevention and treatment services to MSM [22], but the preferences for and comfort in disclosing same-sex behavior to various types of HCPs should be taken into consideration. In general, providing appropriate training for HCPs and creating LGBT-friendly clinical settings may be effective strategies to facilitate the disclosures of same-sex behavior. In China, we suggest that more professional training on effective confidentiality protection and advanced communication skills should be provided to HCPs, especially for communication on sensitive topics [10]. This may be piloted in hospitals or CBOs which are LGBT-friendly or have close links with MSM, and then gradually extended to general HCPs. Considering the current popularity of social media among Chinese MSM, it may be helpful to invite prominent clinicians or public health experts to use social media platforms to highlight the importance of same-sex disclosure to HCPs and to demonstrate the friendliness and confidentiality of the consultation environment for sexual minority groups.

In our study, disclosure of same-sex behavior to any type of HCPs was not significantly associated with a greater likelihood of having ever been tested for HIV after controlling for potential confounding variables including sexual behaviors. This may be because we only investigated having ever been tested for HIV and did not take recent or repeated testing into consideration, and most of the participants recruited from this CBO had also been tested HIV. Several previous studies reported that disclosure to HCPs was associated with ever testing, recent testing, and repeated testing behavior in MSM [10]. Existing studies have also indicated greater healthcare service utilization following HIV testing, such as sexually transmitted infections screening and vaccine uptake, which are part of a panel of healthcare services recommended for MSM [31].

Disclosure of same-sex behavior to clinicians was associated with higher PrEP awareness, which was consistent with two previous studies conducted in the United States [24, 25]. This finding highlights the need to promote patient-provider communication on sexual behaviors and PrEP. The PrEP awareness rate (51%) in the present Chinese MSM sample was much lower than the rates reported by MSM living in other countries (e.g., 81% in the United States [32], 80% in Canada [33]). HCPs are often identified as preferred sources for PrEP by MSM [25], and this low PrEP awareness in our sample highlights the need to enhance the knowledge and skills of HCPs to discuss PrEP with key Chinese populations at risk of HIV. However, the low PrEP awareness rate in our study sample may also be attributed to the fact that PrEP was not yet available in China at the time of the survey.

The disclosure of same-sex behavior to HCPs, regardless of the type of provider, was not associated with an increased awareness of immediate ART or U=U, possibly because the present sample included very few (3%) HIV-positive participants. During routine practice in China, HCPs focus their discussion on safe sex practices when someone is tested negative for HIV, while they focus on ART and U=U when someone is tested positive for HIV [34]. The particularly low level of awareness of U=U (much lower than the level of awareness reported in Western countries [35, 36]) indicates a major information gap in this sample of Chinese MSM. Thus, we recommend that all Chinese MSM regardless of their HIV testing result should be provided a comprehensive information kit containing information on safe sex practices, immediate ART, and U=U. In addition, fears of HIV infection are difficult to ease, and people may remain doubtful about the reliability of the U=U strategy in real life due to the intangibility of measures of viral load [37]. However, awareness of U=U may gradually increase as more people living with HIV are covered by this strategy and share their experiences with others [37].

The present study is subject to several limitations. First, participants were recruited through a CBO located in an urban area, and participants successfully recruited for this study tended to be young and highly educated. In addition, given that the CBO is LGBT-friendly, MSM who were not clients of this CBO may exhibit different characteristics compared to the present sample. Therefore, our findings may not be generalizable to older and less-educated MSM, to MSM living in rural areas, or to MSM who are not clients of LGBT-friendly CBO. Second, due to the small number of HIV-positive participants in the present study, we were unable to perform a subgroup analysis by HIV status, which may be a potential moderator of the association between same-sex behavior disclosure to HCPs and outcomes. Third, there may have been reporting bias or social desirability bias due to stigma and discrimination towards same-sex behavior. Fourth, due to the cross-sectional nature of the data, we were unable to make casual interpretations. Fifth, we did not collect information on the characteristics of the HCPs that might also influence disclosure behavior and patient outcomes. Finally, we were unable to determine the factors or mechanisms that may drive MSM to disclose their same-sex behavior to one type of HCP but not to another type.

Despite these limitations, this is one of the first studies to explore whether same-sex behavior disclosure to HCPs is associated with three most recent and significant HIV prevention and treatment strategies used globally; namely, immediate ART, U=U, and PrEP. We also divided the HCPs to three types (hospital clinicians, CBO peer educators, and CDC public health specialist). This clear picture of the rates of disclosure to these different types of HCPs deepens our understanding of disclosure patterns and will aid the tailoring of specific HIV prevention and treatment programmes for MSM.

## Conclusions

The rates of same-sex behavior disclosure by MSM varied according to the type of HCPs, and were generally low to clinicians, high to peer educators, and medium to public health specialists. Disclosure to clinicians was associated with greater awareness of PrEP, but not awareness of immediate ART or U=U. Disclosure to HCPs (any type) seems not associated with higher lifetime HIV testing rates, awareness of immediate ART or U=U. In addition, the rates of U=U and PrEP awareness were unexpectedly low, indicating a huge information gap in this young, well-educated MSM sample with a predominantly negative or unknown HIV infection status.

## Data Availability

Request can be sent to the corresponding author.
